# Effect of adding a superficial parasternal intercostal plane block to the serratus anterior plane block on postoperative analgesic outcomes in breast cancer surgery: a prospective, randomized, double-blind trial

**DOI:** 10.1007/s00423-026-04065-8

**Published:** 2026-05-07

**Authors:** Fazil Ahmet Akbulut, Hüsnü Kürşad, Muhammed Emin Sözüak, Ali Ahiskalioglu

**Affiliations:** 1 Department of Anesthesiology and Reanimation, Faculty of Medicine, Erzincan Binali Yildirim University, Erzincan, Türkiye Turkey; 2 Department of Anesthesiology and Reanimation, Faculty of Medicine, Ataturk University, Erzurum, Türkiye Turkey

**Keywords:** Serratus plane block, Parasternal intercostal plane block, Regional anesthesia, Postoperative pain, Breast surgery

## Abstract

**Background:**

Breast cancer surgery is frequently associated with moderate to severe postoperative pain. While the serratus anterior plane block (SAPB) provides effective anterolateral chest wall analgesia, it may inadequately cover the anterior intercostal nerve branches. This study aimed to determine whether adding a superficial parasternal intercostal plane block to SAPB improves postoperative opioid consumption, pain scores, and dermatomal sensory coverage.

**Methods:**

In this prospective, randomized, double-blind trial, 64 patients aged 18–65 years undergoing elective breast surgery were randomly allocated into two groups. Group S (*n* = 32) received a preoperative SAPB with 30 mL of 0·25% bupivacaine combined with a parasternal injection of 10 mL saline. Group S + P (*n* = 32) received a SAPB with 30 mL of 0·25% bupivacaine plus a parasternal block with 10 mL of 0·25% bupivacaine. All patients were managed with an identical multimodal postoperative analgesia protocol. The primary outcome was postoperative opioid consumption. Secondary outcomes included visual analogue scale (VAS) pain scores, dermatomal sensory analysis and side effects.

**Results:**

Baseline demographic characteristics and surgical variables were comparable between groups (*p* > 0·05). Sensory blockade of the T3, T4, and T5 dermatomes was significantly more frequent in the S + P group than in the S group (*p* < 0·05). However, postoperative VAS pain scores at rest and during movement, as well as total opioid consumption, were comparable between groups (*p* > 0·05).

**Conclusions:**

Although the addition of a superficial parasternal intercostal plane block to SAPB resulted in wider anterior chest wall dermatomal coverage, it did not confer additional benefit in terms of postoperative pain scores or opioid consumption under a multimodal analgesia regimen. These findings suggest that SAPB-based strategies, when combined with systemic analgesics, may provide sufficient postoperative analgesia in patients undergoing mastectomy.

## Introduction

Breast cancer is the most frequently diagnosed malignancy among women worldwide, and breast surgery constitutes one of the most performed oncological procedures [1, 2]. Despite advances in surgical and anesthetic techniques, moderate to severe postoperative pain is reported by 35–46% of patients following breast surgery [3]. Effective management of acute postoperative pain is essential to facilitate early mobilisation, enhance recovery, and reduce hospital stay. Moreover, inadequately controlled acute pain has been identified as an important risk factor for the development of chronic postoperative pain, with a negative impact on long-term quality of life [4].

Current postoperative pain management protocols relies on multimodal analgesic strategies, combining systemic analgesics with regional anesthesia techniques [5]. Regional analgesia has been shown to reduce postoperative opioid requirements and opioid-related adverse effects [6]. When administered before a predictable noxious stimulus, regional techniques may exert pre-emptive analgesic effects, contributing to improved early recovery, reduced postoperative pain intensity, and decreased systemic analgesic consumption [7].

Thoracic epidural analgesia and paravertebral block have traditionally been regarded as gold standard techniques for breast surgery analgesia. However, their use may be limited by technical complexity, the risk of complications such as hypotension, bradycardia, nerve injury, and haematoma formation, as well as restricted applicability in patients receiving antiplatelet or anticoagulant therapy [8]. With the widespread adoption of ultrasound guidance in anesthetic practice, several interfascial plane blocks have been introduced as safer and more practical alternatives. Techniques such as the erector spinae plane block, pectoral nerve blocks (PECS I–II), serratus anterior plane block (SAPB), and parasternal blocks have increasingly been incorporated into multimodal analgesia protocols for breast surgery [9].

The serratus anterior plane block (SAPB), performed between the latissimus dorsi and serratus anterior muscles, provides effective analgesia of the anterolateral chest wall, typically covering the T2–T9 dermatomes [10]. However, SAPB does not reliably block the anterior cutaneous branches of the intercostal nerves responsible for sensation of the anteromedial chest wall. In this context, a parasternal intercostal plane block, applied adjacent to the sternum between the pectoralis major and intercostal muscles, may complement SAPB by extending sensory coverage to the anterior chest wall.

This study aimed to evaluate whether the addition of a parasternal intercostal plane block to the SAPB as part of a multimodal analgesia regimen affects postoperative pain scores, opioid consumption, and dermatomal sensory distribution in patients undergoing breast surgery. The primary outcomes were 24-hour postoperative visual analog scale (VAS) pain scores. Secondary outcomes included dermatomal sensory examination, total postoperative fentanyl consumption, and opioid-related adverse effects.

## Methods

This prospective, randomized, double-blind study was conducted following approval from the Atatürk University Faculty of Medicine Ethics Committee (31.03.2022-3/14). The trial was registered with ClinicalTrials.gov (ID: NCT05911373) and carried out between June 2023 and January 2024.

After approval from the Local Ethics Committee of the Atatürk University Faculty of Medicine, patients aged 18–65 years with American Society of Anesthesiologists (ASA) physical status I–II scheduled for unilateral mastectomy or modified radical mastectomy were screened for eligibility. Exclusion criteria included significant cardiovascular disease, hepatic dysfunction, body mass index ≥ 35 kg/m², coagulopathy or use of anticoagulant therapy, infection at the injection site, inability to cooperate, known allergy to study drugs, and refusal to participate.

Eligible patients were randomly allocated in a 1:1 ratio to one of two groups (34 patients per group) using a computer-generated randomisation sequence (Microsoft Excel RAND function; Microsoft, Redmond, WA, USA). Group allocation was concealed. Study medications were prepared by an investigator not involved in anaesthetic management or data collection. Both the anaesthesiologists responsible for perioperative care and the investigators collecting outcome data were blinded to group allocation. All blocks were performed by the same experienced anaesthesiologist, and all surgical procedures were conducted by the same surgical team.

After written informed consent was obtained, patients were transferred to the block room prior to surgery. Standard monitoring, including electrocardiography, pulse oximetry, and non-invasive blood pressure, was applied.

All blocks were performed in awake patients in the block room prior to induction of general anesthesia, in accordance with our routine clinical practice and to allow dermatomal sensory assessment. No additional sedation was administered before block placement. Local infiltration with a small volume of local anesthetic was applied at the needle insertion site to improve patient comfort.

### Group S (SAPB group)

With the patient in the lateral decubitus position, the skin and a high-frequency linear ultrasound probe (Esaote MyLab X7, CA631, London, UK) were prepared under sterile conditions. The probe was positioned obliquely medial to the mid-axillary line at the level of the fourth and fifth ribs to identify the latissimus dorsi and serratus anterior muscles. Using an in-plane technique, a 22-gauge, 100-mm needle (Stimuplex^®^, B. Braun, Melsungen, Germany) was advanced beneath the latissimus dorsi muscle. After hydrodissection with 2 mL saline, 30 mL of 0·25% bupivacaine with epinephrine (5 µg/mL) was injected between the latissimus dorsi and serratus anterior muscles. Epinephrine was added to the local anesthetic solution to reduce systemic absorption and prolong the duration of action.

Subsequently, with the patient in the supine position, a parasternal injection of 10 mL 0·9% saline was performed under ultrasound guidance into the interfascial plane between the pectoralis major and intercostal muscles at the level of the fourth and fifth ribs, adjacent to the sternum.

### Group S + P (SAPB plus parasternal block group)

Patients in this group received the same SAPB technique. In addition, a parasternal intercostal plane block was performed using 10 mL of 0·25% bupivacaine injected under ultrasound guidance into the interfascial plane between the pectoralis major and intercostal muscles at the fourth–fifth rib level.

### Dermatomal assessment

Dermatomal sensory assessment was performed 30 min after block administration using a cold sensation test, whereby loss or reduction of cold perception, compared with the contralateral side was recorded as sensory blockade. The anterior hemithorax was divided by the midclavicular line, and sensory involvement of medial and lateral regions was recorded for each dermatome.

### Anaesthetic management

All patients received standardised general anaesthesia. Anaesthesia was induced with midazolam (0·1 mg/kg), propofol (2 mg/kg), and rocuronium (0·6 mg/kg). Following induction, all patients received fentanyl (1–2 µg/kg). Additional intraoperative fentanyl was administered based on haemodynamic responses (increase in heart rate or mean arterial pressure > 20% from baseline. Anaesthesia was maintained with 1–2% sevoflurane in a 50% oxygen–air mixture. Neuromuscular blockade was reversed with sugammadex (2–3 mg/kg). Patients who were haemodynamically stable and responsive were transferred to the recovery unit and discharged to the ward once a Modified Aldrete score ≥ 9 was achieved. The same anaesthesia protocol and criteria were applied to all patients.

### Postoperative analgesia protocol

All patients received intravenous paracetamol (1000 mg) and dexketoprofen (50 mg) approximately 30 min before the end of surgery. Postoperatively, paracetamol (1000 mg every 8 h) and dexketoprofen (50 mg every 12 h) were continued. Patient-controlled analgesia (PCA) with fentanyl (10 µg/mL; bolus 25 µg; lockout interval 15 min; no basal infusion) was initiated in the recovery unit and maintained for 24 h.

### Postoperative follow-up

Pain during activity and rest after surgery was assessed using a visual analog scale (VAS) 1, 2, 4, 8, 12, and 24 h after surgery. The patient was asked to cough to assess pain during activity. Time to first analgesic request, total opioid consumption, nausea and vomiting, sedation, pruritus, drug-related adverse effects, and block-related complications were recorded. Intravenous tramadol (100 mg) was administered as rescue analgesia in patients with a VAS score ≥ 4 despite fentanyl PCA.

### Sample size and statistical analysis

Based on previous data reported by Jain et al. [11], showing a 24-hour fentanyl consumption of 415 ± 182 µg following SAPB, a sample size of 28 patients per group was calculated to detect a 30% reduction in opioid consumption, with an effect size of 0·68, α error of 0·05, and power of 80%. To account for potential dropouts, 32 patients were included in each group.

Statistical analysis was performed using IBM SPSS version 22. Normally distributed continuous variables were expressed as mean (SD), non-normally distributed variables as median (IQR), and categorical variables as number (%). Student’s t test or Mann–Whitney U test was used for continuous variables as appropriate, and χ² test for categorical variables. A p value < 0·05 was considered statistically significant.

## Results

**Fig. 1:**
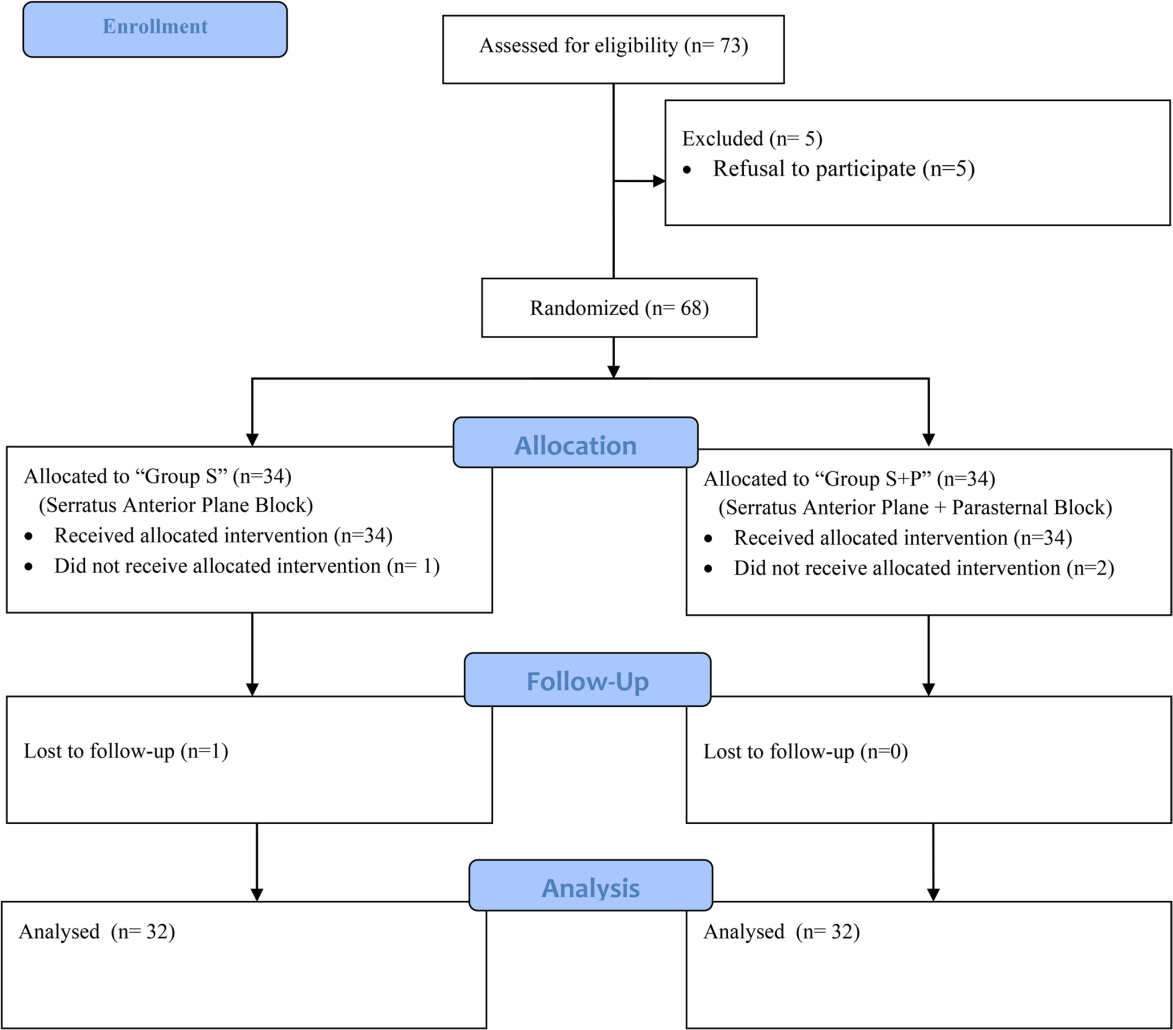
Note: This data is mandatory, please provide

During the study period, 73 patients were assessed for eligibility. Five patients declined to participate in the study. Four patients were excluded from the final analysis: three declined participation at the block stage and one required postoperative reoperation for haematoma drainage. Consequently, data from 64 patients (32 per group) were analysed. The CONSORT flow diagram is presented in Fig. 1.

Baseline demographic characteristics, surgical type, incision length, anaesthesia duration, and operative time were comparable between the two groups, with no statistically significant differences observed (Table 1; all *p* > 0·05).

**Table 1:** Demographic characteristics of the study

	Group S*n*=32	Group S+P*n*=32	*P* value
Age(year)	55.09±11.73	53.47±11.74	0.670^a^
Height (metres)	1.60 (1.58–1.64)	1.60 (1.58–1.63)	0.766^b^
Weight (kg)	75 (70–80)	78 (70–80)	0.530^b^
BMI (kg-m^2^)	28.53 ± 3.60	29.10 ± 3.60	0.509^a^
ASA (I-II)	19 − 13	15–17	0.453^c^
Total anesthesia duration (min)	117.50 (92.50-152.50)	107.50 (90–120)	0.247^b^
Total surgical time (min)	92.50 (75-117.50)	85 (75–100)	0.374^b^
Incision size (cm)	24 (20–25)	22 (20-27.50)	0.967^b^
Surgical type (SM/MRM)	20/12	21/11	0.794^c^

All data mean ± SD median (25%-75% percentile) or number, SM: Simple mastectomy, MRM: modified radical mastectomy,

^a^ Independent t-test,

^b^ Mann-Whitney u test,

^c^ Chi-square test

Dermatomal sensory assessment of the anterior chest wall revealed significantly greater medial anterior hemithorax involvement in the S + P group at the T3, T4, and T5 dermatomes compared with the S group (*p* = 0·016, *p* < 0·001, and *p* = 0·030, respectively; Table 2). No significant differences were identified between groups in dermatomal involvement of the lateral anterior chest wall across any thoracic level (all *p* > 0·05).

**Table 2:** Dermatomal involvement of the medial-lateral of anterior chest wall

	Group S(*n*=32)	Group S+P(*n*=32)	*P* value
Medial of anterior hemithorax			
T1	11 (%34.37)	15 (%46.87)	0.309
T2	21 (%65.62)	26 (%81.25)	0.157
T3	21 (%65.62)	29 (%90.62)	**0.016**
T4	21 (%65.62)	32 (%100)	**< 0.001**
T5	22 (%68.75)	29 (%90.62)	**0.030**
T6	20 (%62.50)	26 (%81.25)	0.095
Lateral of anterior hemithorax			
T1	13 (%40.60)	14 (%43.75)	0.800
T2	26 (%81.25)	23 (%71.87)	0.376
T3	29 (%90.62)	30 (%93.75)	0.641
T4	30 (%93.75)	31 (%96.87)	0.554
T5	30 (%93.75)	29 (%90.62)	0.641
T6	25 (%78.12)	26 (%81.25)	0.756

Chi-square test was used for statistical analysis. T: Thoracic dermatome area

Postoperative opioid consumption did not differ significantly between groups at any time interval during the first 24 h (Table 3). Total fentanyl consumption was comparable between the S group and the S + P group (median [IQR]: 200 [50–375] µg vs. 125 [100–237·5] µg; *p* = 0·798). Similarly, the requirement for rescue analgesia with tramadol did not differ significantly between groups (*p* = 0·171).

**Table 3:** Total fentanyl consumption in microgram units by hours

First 4 h	Group S (*n* = 32)	Group S + *P* (*n* = 32)	*P* value
	62.50 (25 -87.50)	50 (25–75)	0.753^a^
4–8 h	50 (0–75)	25 (0-62.50)	0.726^a^
8–12 h	25 (0–75)	25 (0–50)	0.813^a^
12–16 h	25 (0–50)	25 (0–50)	0.768^a^
16–24 h	25 (0-62.50)	25 (0-37.50)	0.590^a^
Total fentanyl consumption	200 (50–375)	125.00 (100-237.50)	0.798^a^
Additional analgesic need (present/absent)	25/7	20/12	0.171^b^

Values are shown as median (25%-75% percentile) or number (n)

^a^ Mann-Whitney U test,

^b^ Chi-square test

Postoperative pain scores assessed using the visual analogue scale (VAS) at rest and during movement at 1, 2, 4, 8, 12, and 24 h were similar between groups, with no statistically significant differences detected at any time point (Table 4; all *p* > 0·05).

**Table 4:** VAS scores

	Group S(*n*=32)	Group S+P(*n*=32)	*P* value
VAS scores resting			
PACU	1.00 (00-2.37)	2.00 (00–4.00)	0.051
1.hour	1.50 (1.00-2.50)	3.00 (1.00–4.00)	0.056
2.hour	1.50 (0.50-2.00)	1.00 (1.00-2.50)	0.934
4.hour	1.00 (00–2.00)	1.00 (0.50-2.00)	0.358
8.hour	1.00 (00–2.00)	1.00 (1,00–2,00)	0.161
12.hour	1.00 (00–1.00)	1.00 (00–2,00)	0.559
24.hour	1.00 (00–1.00)	1.00 (00–1.00)	0.708
VAS scores during activity			
PACU	1.00 (00-2.50)	2,50(00/5,00)	0.072
1.hour	2.00 (0.50-3.00)	3.00 (1.00-4.50)	0.083
2.hour	2.00 (1-00-3.00)	2.00 (1.00–3.00)	0.913
4.hour	1.50 (1.00–3.00)	2.00 (1.00–3.00)	0.305
8.hour	1.00 (1.00-2.50)	2.00 (1.00–3.00)	0.123
12.hour	1.00 (0.50-2.00)	1.50 (00–2.00)	0.652
24.hour	1.00 (00–2.00)	2.00 (1.00–2.00)	0.468

Values are shown as rest and active VAS scores and the median (25th–75th percentile)

The Mann-Whitney U test was used for statistical analysis

The incidence of opioid-related adverse effects, including nausea, vomiting, requirement for anti-emetic therapy, constipation, pruritus, urinary retention, and dry mouth, was low and comparable between groups (Table 5). No statistically significant differences were observed in the overall frequency of opioid-related side effects (all *p* > 0·05). No complications related to the block were observed.

**Table 5:** Opioid-related side effects according to groups

	Group S(*n*=32)	Group S+P(*n*=32)	*P* value
Nausea	6 (%18.75)	4 (%12.5)	0.49
Vomiting	2 (%6.25)	2 (%6.25)	1
Anti-emetic need	1 (%3.12)	1 (%3.12)	1
Constipation	8 (%25)	3 (%9.37)	0.098
Itching	2 (%6.25)	2 (%6.25)	1
Urinary retention	0	0	NS
Dry mouth	4 (%12.5)	2 (%6.25)	0.391

Chi-square test was used for statistical analysis. *NS* Non-significant

## Discussion

The present study demonstrates that, within a multimodal analgesic regimen, effective postoperative analgesia can be achieved after mastectomy even in the absence of a parasternal block added to a pre-emptively administered serratus anterior plane block (SAPB). Although the addition of a parasternal block resulted in wider sensory coverage of the anterior hemithorax, this did not translate into clinically meaningful differences in postoperative pain scores, opioid consumption, or the need for rescue analgesics.

Enhanced recovery after surgery (ERAS) protocols consistently emphasise multimodal analgesia as a cornerstone of perioperative care, with the primary aim of minimising opioid exposure while facilitating early recovery and discharge. To this end, combinations of paracetamol, non-steroidal anti-inflammatory drugs, magnesium, ketamine, α₂-agonists, and regional anaesthesia techniques are commonly employed [12]. In line with these principles, all patients in our study received a standardised multimodal analgesic regimen incorporating pre-emptive regional blocks alongside systemic non-opioid analgesics, which likely contributed to the overall satisfactory postoperative analgesic outcomes observed in both groups.

In recent years, interfascial plane blocks such as the erector spinae plane block, pectoral nerve blocks (PECS I–II), and SAPB have been increasingly adopted as alternatives to neuraxial techniques for breast surgery. This shift reflects concerns about the technical complexity and potential complications associated with thoracic epidural and paravertebral blocks, as well as the widespread availability of ultrasound guidance. Although the anterior cutaneous branches of the intercostal nerves are not directly blocked in these methods, numerous studies have reported that these interfascial techniques provide effective postoperative analgesia.

Anatomically, sensory innervation of the anteromedial chest wall is mediated primarily by the anterior branches of the intercostal nerves, which are not reliably blocked by SAPB [13]. For instance, a prospective study by Pascarella et al. 2023 parasternal block cardiac surgery in patients undergoing cardiac surgery demonstrated that the addition of a parasternal block was associated with reduced intraoperative opioid consumption, shorter time to extubation, and improved postoperative respiratory performance [14]. However, despite these favourable intraoperative and functional outcomes, postoperative opioid consumption remained comparable between groups. These findings are in line with our results, in which improved anterior dermatomal coverage with the addition of a parasternal block did not translate into a measurable reduction in postoperative pain scores or opioid requirements. On this basis, parasternal intercostal plane blocks have been proposed to complement SAPB or PECS blocks by extending sensory coverage medially. Fusco and colleagues demonstrated the feasibility of combining parasternal block with SAPB to facilitate breast surgery under sedation, supporting the theoretical benefit of anterior intercostal nerve blockade [15]. However, as mentioned earlier, several clinical studies evaluating SAPB or PECS blocks that do not specifically target the anterior branches of the intercostal nerves have reported favourable analgesic results [16, 17]. Similarly, Hozien et al. found no significant differences in postoperative pain scores or opioid consumption when comparing SAPB alone with SAPB combined with a pectointercostal plane block [18]. These findings highlight the ongoing uncertainty regarding the clinical necessity of routinely blocking the anterior intercostal nerve branches in breast surgery.

In this context, our study adds novel data by directly comparing SAPB alone with SAPB combined with a parasternal block, while also providing a detailed dermatomal analysis of medial and lateral anterior chest wall involvement. Although parasternal block significantly increased sensory blockade in the T3–T5 dermatomes of the medial anterior hemithorax, this anatomical advantage did not confer additional analgesic benefit. Interestingly, some degree of medial dermatomal involvement was also observed in patients receiving SAPB alone, which may reflect local anaesthetic spread from the serratus–latissimus dorsi fascial plane toward the intercostal space, resulting in partial blockade of the main intercostal nerves. This situation may be due to the high volume we are supplying to SAPB. Cadaveric studies are warranted to further elucidate this potential mechanism.

In a cadaver study conducted by Mayes et al., it was demonstrated that the serratus plane muscle does not extend into the intercostal space and therefore does not anesthetize the intercostal nerve. This study also showed that the viscosity of the solution applied to the interfascial space can alter its spread. They reported that the analgesic effect on the anterior chest wall may be due to the absorption of the local anesthetic agent and its passage into the systemic circulation [19]. In our study, the use of a relatively high volume (30 mL) of bupivacaine for SAPB may have contributed to broader sensory effects, potentially explaining the comparable analgesic outcomes between groups. Consistent with this, dermatomal involvement of the lateral anterior hemithorax did not differ significantly between groups.

While some studies advocate the routine addition of parasternal blocks to enhance anterior chest wall analgesia [20], our findings suggest that, when SAPB is administered pre-emptively using an adequate volume of local anaesthetic within a comprehensive multimodal analgesic regimen, the incremental clinical benefit of parasternal blockade may be limited. This interpretation is consistent with previous studies showing that serratus anterior plane block (SAPB) on its own is associated with a significant reduction in postoperative opioid requirements, while adding anterior intercostal or parasternal blocks has not consistently provided additional analgesic benefit over SAPB alone [21].

Some limitations of this study should be acknowledged. Firstly, the sample size may not be sufficient to detect rare adverse events. Secondly, dermatomal assessment was limited by predefined examination boundaries, and intraoperative opioid consumption was not recorded. Third; the lack of long-term follow-up, which precludes any conclusions regarding the potential role of parasternal intercostal plane block in the prevention of chronic postoperative pain. Previous studies, such as that by Fujii et al. PECS II vs. serratus plane chronic pain, have demonstrated that differences between regional anesthesia techniques may become more apparent over time [22]. Future studies incorporating extended follow-up are therefore warranted to better evaluate the impact of these techniques on chronic pain outcomes. Additionally, the combined use of multiple systemic analgesics may have reduced potential differences between regional techniques; however, this approach reflects ERAS-based clinical practice and is consistent with the study’s pragmatic design. Another limitation of this study is the absence of a validated postoperative recovery assessment tool such as the QoR-15 questionnaire. Inclusion of such patient-reported outcome measures could have provided a more comprehensive evaluation of functional recovery, including respiratory comfort and overall well-being. Future studies should consider integrating these measures to better capture the broader clinical impact of regional anesthesia techniques. Lastly; the use of a sevoflurane-based anesthesia protocol and midazolam premedication may have influenced secondary outcomes such as nausea and vomiting. In addition, the use of inhalational anesthesia may have limited the ability to fully isolate opioid-related adverse effects. Future studies employing propofol-based total intravenous anesthesia techniques may allow for a more precise evaluation of opioid-related outcomes.

It should be noted that postoperative opioid consumption in both groups was lower than anticipated in the initial power calculation, likely reflecting the effectiveness of the multimodal analgesic regimen. As a result, the study may have been underpowered to detect small but clinically relevant differences between groups. Therefore, the absence of statistically significant differences should be interpreted with caution and should not be considered as definitive evidence of no effect. In addition, although not statistically significant, there was a trend toward improved early postoperative pain scores in the parasternal block group, which may become more apparent in studies with larger sample sizes.

In conclusion, our findings suggest that, for patients undergoing breast surgery, pre-emptive administration of a high-volume serratus anterior plane block as part of a multimodal analgesic strategy may provide effective postoperative analgesia without the need for additional parasternal blockade to target the anterior intercostal nerve branches.

## Data Availability

No datasets were generated or analysed during the current study.
